# Agrivoltaic panel shading influences soil nutrient dynamics and microbial community structure in a wheat-cultivated field

**DOI:** 10.1371/journal.pone.0352589

**Published:** 2026-07-14

**Authors:** Claudia Chiodi, Federico Gavinelli, Shunlei Li, Massimo Cagnin, Davide Del Todesco Frisone, Sara Remelli, Aldo Dal Prà, Giuseppe Concheri, Andrea Squartini, Asia Paone, Cristina Menta, Piergiorgio Stevanato

**Affiliations:** 1 Department of Agronomy, Food, Natural Resources, Animals and Environment, University of Padova, Padova, Italy; 2 Department of Chemistry, Life Sciences and Environmental Sustainability, University of Parma, Parma, Italy; 3 Institute of BioEconomy-National Research Council, Florence, Italy; 4 REM Tec S.R.L., Mantova, Italy; Sultan Qaboos University College of Science, OMAN

## Abstract

Agrivoltaic systems (AV), which combine photovoltaic panels with crop cultivation, represent a dual land use strategy to address food and energy security challenges under growing population pressures. However, the effects of AV systems on soil biogeochemical processes and microbial communities remain insufficiently characterized. This study investigated the effects of AV trackers with varying Ground Coverage Ratio (GCR) on soil physicochemical properties, and microbial community composition and diversity at two time points (T1 and T2). Field experiments were conducted in a wheat-cultivated field using two AV configurations: standard (STV1, GCR = 13%) and extended (STV2, GCR = 41%) trackers, compared to a full-sun control (CI). Soil chemical analysis performed using a combustion analyzer showed that organic carbon, total nitrogen, Olsen phosphorus, and exchangeable calcium, potassium, and magnesium were all increased under STV2 compared to both STV1 and the control; this overall enrichment was further confirmed by ICP-OES-based elemental profiling. Amplicon sequencing of the 16S rRNA and ITS1 regions revealed reduced bacterial and fungal diversity under STV2. Significant taxonomic shifts in bacterial and fungal communities were also observed at both phylum and genus levels relative to the control. These findings suggest that extended shading from STV2 altered soil chemical properties while restructuring microbial communities, reducing diversity and favoring copiotrophic taxa, especially at T1. Overall, the results underscore the central role of agrivoltaic design, specifically shading intensity and GCR, in shaping belowground processes with relevant implications for soil health and the agroecological performance of dual land use energy–food systems.

## 1. Introduction

Global agriculture systems are facing increasing challenges, including resource constraints, climate variability, pressure on land use, and the growing vulnerability of energy markets. Ensuring long-term sustainability and resilience requires innovative strategies to integrate food and energy production while minimizing environmental impact.

Within this context, renewable energy development has emerged as a key strategy for ensuring long-term energy security [1]. Among emerging approaches, agrivoltaics (AV), a land-sharing system that combines photovoltaic energy generation with crop cultivation, has attracted significant attention for its potential to optimize land use, increase system productivity, and reduce environmental impacts [2,3]. The physical presence of photovoltaic (PV) panels influences local microclimatic conditions by modifying solar radiation flux, wind speed, and even air temperature beneath the structures  [4–6]. Shading also alters rainfall redistribution at soil, concentrating water at panel edges and generating heterogeneous soil water distribution and localized water storage [6–8]. By lowering potential evaporation directly under panels, these systems reduce drought stress, enhance soil water availability, and limit water loss in arid climates [9–11]. In addition, appropriate shading can decrease evapotranspiration by reducing stomatal opening, thereby improving crop water-use efficiency, although species-specific responses remain evident. PV panels buffer soil temperature fluctuations: they cool the soil during hot daytime conditions while reducing nighttime cooling in autumn and winter, with reported variations of up to 10 °C compared to open-field soil [6,9].

Such regulation of soil temperature and moisture, combined with increased relative humidity under panels [2,12], has been shown to enhance crop performance under heat and drought stress, by reducing heat stress, increasing water-use efficiency, and, in some cases, stabilizing yields under adverse climatic conditions [8,10,13]. However, crop responses vary with species, management practices, and panel configuration. Evidence from vegetable systems highlighted variability: in lettuce, the most frequently studied species, shading has produced outcomes ranging from substantial yield reductions to marked increases, often depending on season and variety [14,15]. Tomatoes likewise show contrasting responses, with some trials reporting reduced fruit set under diminished light, while others highlight improved fruit size and physiological adaptation under partial shade [13,16]. Cereals were less investigated: maize has demonstrated tolerance to moderate shading, with slight yield gains under low panel density due to reduced evaporative losses, but sharp declines at higher coverage [17]. Wheat showed contradictory results as well: increases in vegetative biomass have been observed, but grain yields often decline, particularly in terms of grain weight [2,18].

The same microclimatic modifications that improve crop performance also influence soil biological functioning, particularly the activity and composition of microbial communities. Soil microorganisms dynamically respond to shifts in temperature, moisture, and root exudation patterns, which are shaped by AV design and shading [19–21]. In fact, panel-induced heterogeneity generates diverse soil microhabitats that are crucial for sustaining microbial diversity [22]. Panels alter key abiotic factors, thereby restructuring microbial biomass, richness, and composition [23,24]. For example, shading can increase total microbial biomass by 20–30%, while reducing alpha diversity indices due to the declines in light-dependent taxa and the competitive release of shade-adapted groups [24,25]. Beyond microclimate, panel coverage also affects nutrient dynamics, with C and N storage decreasing by up to 61% and 50%, respectively, resulting in cascading effects on microbial functional groups. These effects include increases in Proteobacteria and reductions in Acidobacteria [26,27].

Existing studies often report contrasting results, as crop and soil responses are shaped not only by species and local environmental conditions, but also by the design of the AV installation itself [23,28]. Different technological solutions have been developed to integrate photovoltaic power generation and crop cultivation. Interspersed PV arrays place panels between crop rows, whereas overhead systems can either be greenhouse-mounted, replacing part of the transparent cover, or stilt-mounted above open-field crops [29]. Panels may be static or dynamic, the latter adjusting their tilt to balance energy generation with crop light requirements [9]. Moreover, the ground coverage ratio (GCR), the proportion of soil surface occupied by PV modules relative to the total cultivated area, has emerged as a key parameter linking energy output and agricultural productivity [30]. Increasing GCR generally enhances energy yield but can negatively affect crop performance by reducing light availability [30]. However, while the relationship between GCR and crop yield has been explored, its implications for soil physical, chemical, and biological functioning remain largely overlooked. This knowledge gap is particularly critical for temperate cereal-based agroecosystems, where soil health is essential for long-term productivity.

This study investigates the impact of different agrivoltaic (AV) ground coverage ratios on soil properties and microbial communities in a wheat-based system in Northern Italy. We hypothesize that increasing AV GCR modify soil physicochemical characteristics and, in turn, influence the composition and structure of soil microbial communities. The research specifically aims to evaluate: (i) how AV GCR can influence soil physicochemical characteristics and (ii) whether AV installations alter the structure composition of microbial communities as an agroecosystem service. Addressing these aspects provides insights into the potential of AV systems to sustain not only energy generation and crop yield, but also the long-term integrity and health of soils.

## 2. Materials and methods

### 2.1. Study area

The study area is located in Borgo Virgilio (Mantova Province, northern Italy; 45°05’38.19”N, 10°47’22.92”E) and hosts the agrivoltaic system investigated in this study. Access to the site was granted by REM Tec S.R.L., which manages the facility. The system was installed in 2011, and it spans a total surface area of 11.42 hectares, of which 13% (1.30 hectares) is shaded. The photovoltaic system has a nominal capacity of 2150.4 kW and consists of 768 dual-axis trackers (BIAXIAL TRACKERS 3D-T1.0) supporting 7680 polycrystalline silicon (Poly) panels, each rated at 280 Wp. The installed power density is approximately 188 kWp/ha. The dual-axis tracking system automatically adjusts the panels’ inclination angles in response to the sun’s position, optimizing solar energy capture. The primary (central) axis rotates from 50° (morning) to 140° (afternoon), passing through 90° (horizontal), while the secondary axis shifts from an initial position of 90° (flat) up to 135°-140°, and down to 50° by evening. Regarding structural specifications, the maximum clearance beneath the panels in their safety position is 4.5 m. In contrast, the minimum ground clearance at the panels’ maximum inclination is approximately 3.20 m [31].

The crop cultivated in the portion of the field considered for this study was Durum wheat (*Triticum durum* Desf.) Levante (Syngenta Italia, Milano, Italy); seeds were sown at a density of 350 seeds m^−2^. The crop previously cultivated was Italian ryegrass (it was mechanically destroyed by ploughing). Pre-sowing harrowing was performed one week prior to sowing on October 20^th^, 2023, using a combined row seeder (Nina 250–400, Maschio Gaspardo, Campodarsego, Padova, Italy). Fertilization rates were 100 kg N ha⁻¹ and 40 kg K ha⁻¹, adjusted based on soil chemical analysis. No irrigation was applied.

According to Soil Taxonomy, the soil is classified as Typic Calciustepts, fine-silty, mixed, mesic (Soil Survey Staff, 2022), corresponding to an Endocalcaric Calcisol (Siltic, Hypereutric) in the WRB system (IUSS Working Group WRB, 2022).

During the study period (March–July 2024), the mean air temperature was 15.1 °C, total rainfall was 815 mm, mean relative humidity was 83.3%, and mean daily global solar radiation was 16.18 MJ m⁻² day⁻¹. The site typically receives 1800–2000 hours of sunlight per year.

### 2.2. Soil sampling and experimental design

Soil was sampled under two agrivoltaic (AV) configurations: extended trackers (STV2) and standard trackers (STV1), and in a full-sun control (CI) condition. Ground cover ratio (GCR) was 41% (STV2) and 13% (STV1).

For each condition, three plots were selected, and three samples per plot were collected from the 0–30 cm layer using a manual auger, for a total of 9 replicates per condition. The 9 plots considered, three for each experimental condition, were randomly distributed along an inter-row corridor designated for the experiment. Each plot had a surface area of 12 x 12 m, for a total tested area of 1,296 m^2^.

Sampling was performed twice with the same sampling design: at the wheat early vegetative stage – March 10^th^ (T1), and at the harvest stage – July 2^nd^ (T2), 2024. Soil samples were air-dried and sieved at 2 mm for analysis.

### 2.3. Soil physicochemical and elemental analyses

The particle size distribution (PSD) was determined for each soil sample using laser diffraction analysis, with Mastersizer 2000 (Malvern Panalytical, Malvern, United Kingdom). Soil pH was measured according to a standardized protocol using a glass electrode in a suspension of soil and ultra-pure water at a 1:2.5 (w/v) ratio. The same suspension was used to measure electrical conductivity (EC). To quantify the total nitrogen (N_tot_) and total carbon (C_tot_) contents, samples were subjected to dry combustion analysis using an Elementar Vario MACRO CNS analyzer (Elementar Analysensysteme GmbH, Hanau, Germany). Organic carbon (C_org_) was specifically assessed by combustion with the Skalar PrimacsSNC−100 analyzer (Skalar Analytical BV, Breda, The Netherlands), after removal of inorganic carbon fractions. The plant-available phosphorus (P) concentration was determined using the Olsen P extraction method [32], suitable for soils with neutral to alkaline pH.

Exchangeable bases (Ca²⁺ , Mg²⁺ , K⁺ , Na⁺) were extracted using 1 M ammonium acetate at pH 7.0. Then, the elemental composition was determined by inductively coupled plasma optical emission spectrometry (ICP-OES), using a Spectro Arcos MV instrument (Spectro Ametek, Kleve, Germany). Quantification was performed using multi-point external calibration curves for each element. Quality assurance and quality control (QA/QC) procedures included the analysis of procedural blanks, replicate measurements, calibration verification standards, and certified reference materials.

### 2.4. Total soil DNA extraction, 16S and ITS metabarcoding

Total genomic DNA was extracted from 250 mg of air-dried soil using the DNeasy PowerSoil Pro Kit (Qiagen GmbH, Hilden, Germany), according to the manufacturer’s protocol. Final elution was performed with nuclease-free, autoclaved double-distilled water (ddH₂O). DNA concentration was quantified using a Qubit Flex fluorometer (Thermo Fisher Scientific, Carlsbad, CA, USA) in combination with the Qubit 1× dsDNA High Sensitivity Assay Kit (Thermo Fisher Scientific), enabling accurate quantification of low-concentration DNA samples.

DNA samples were processed and sequenced at Novogene (Germany) for 16S and ITS rDNA metabarcoding. Briefly, the V4–V5 hypervariable regions of the 16S rRNA gene were amplified using the primer pair GTGCCAGCMGCCGCGGTAA (forward) and CCGTCAATTCCTTTGAGTTT (reverse), while the ITS1 region was amplified using primers CTTGGTCATTTAGAGGAAGTAA (forward) and GCTGCGTTCTTCATCGATGC (reverse). Short-reads sequencing was performed on the Illumina NovaSeq platform to generate 2 × 250 bp paired-end reads.

### 2.5. Data analysis for 16s and ITS metabarcoding

Raw paired-end reads were initially processed by Novogene (UK) using their standard amplicon bioinformatics workflow based on QIIME2. Reads were demultiplexed, merged, and subjected to quality filtering and trimming to obtain high-quality clean tags. Chimeric sequences were removed during this preprocessing stage. Clean reads were subsequently denoised and dereplicated into amplicon sequence variants (ASVs) using DADA2 [33]. Representative ASV sequences were taxonomically classified using QIIME2 classify sklearn plugin [34] with the SILVA 138.1 database for bacterial 16S [35] and the UNITE v9.0 database for fungal ITS [36]. ASVs and their respective count tables were used to compute diversity indices and relative abundance profiles, perform sample clustering, and conduct downstream statistical analyses. Microbiome data analyses were performed using MicrobiomeAnalyst 2.0, with data normalization using Cumulative Sum Scaling (CCS) [37,38]. The suite was used to obtain alpha-diversity metrics, species composition, and beta-diversity estimation. Alpha diversity at the genus level was calculated using Chao1, Shannon, and Simpson indices. Beta diversity of microbial communities at the genus level was assessed using Principal Coordinate Analysis (PCoA) based on Bray-Curtis distances. Differences in community composition were statistically tested using Multivariate Analysis of Variance (PERMANOVA) based on Bray-Curtis distance matrices, including sampling time and experimental condition as fixed factors, with 9999 permutations. When the effect of experimental condition was significant, pairwise PERMANOVA comparisons among conditions were performed with false discovery rate (FDR) correction for multiple testing. Linear Discriminant Analysis Effect Size (LEfSe) was performed to identify genera associated with the different conditions, with an LDA score threshold of 2.0 [39]. Data visualisation was conducted using SRplot [40].

### 2.6. Statistical analyses

Data from three experimental conditions (n = 9 replicates per condition) were analyzed to assess statistical differences. A one-way analysis of variance (ANOVA) was performed, and when significant differences were detected (p < 0.01), pairwise comparisons were conducted using Tukey’s Honestly Significant Difference (HSD) post-hoc test. Statistical significance was set at p < 0.01. This analytical pipeline was applied to evaluate differences in soil physico-chemical properties, elemental composition, and genus- and phylum-level abundances, at both time points.

Differences in alpha-diversity among conditions were assessed using the non-parametric Kruskal–Wallis test. Statistical significance was indicated as follows: ns (not significant), *p-value < 0.1, **p-value < 0.05, ***p-value < 0.01.

A multivariate Canonical Correspondence Analysis (CCA) was conducted to assess the microbial communities concerning physico-chemical and elemental composition as environmental variables. CCA plots were constructed in Statistica v13.5 (TIBCO Software Inc.), using Scaling Type 2 to ensure accurate interpretation of compositional gradients.

## 3. Results

### 3.1. Soil physico-chemical profiles

Stable soil characteristics (clay, silt, sand content, and pH) did not exhibit significant variation across the different agrivoltaic (AV) configurations and the control (Table 1).

**Table 1:** Physicochemical properties across different conditions. Values are expressed as mean ± standard error (n = 9). CI: control; STV1: standard trackers; STV2: extended trackers. Different letters indicate a statistically significant difference in the post-hoc test (p < 0.01) (S1 Table in S1 File).

Parameter	CI	STV1	STV2
Clay (%)	31.78 ± 0.68 a	31.57 ± 0.75 a	31.57 ± 0.59 a
Silt (%)	29.80 ± 0.49 a	30.91 ± 0.62 a	30.68 ± 0.67 a
Sand (%)	38.41 ± 1.02 a	37.52 ± 0.68 a	37.74 ± 0.59 a
N_tot_ (%)	0.10 ± 0.01 b	0.10 ± 0.01 b	0.13 ± 0.00 a
C_tot_ (%)	3.92 ± 0.26 a	3.74 ± 0.16 a	2.76 ± 0.09 b
C_org_ (%)	0.96 ± 0.04 b	0.98 ± 0.05 b	1.17 ± 0.01 a
C/N	10.06 ± 0.23 a	9.63 ± 0.26 ab	9.03 ± 0.12 b
DM	97.02 ± 0.28 a	96.09 ± 0.12 b	96.17 ± 0.12 ab
Olsen P (mg/kg)	16.78 ± 2.97 b	13.31 ± 1.94 b	26.87 ± 0.54 a
pH	8.34 ± 0.02 a	8.44 ± 0.03 a	8.43 ± 0.03 a
EC (mS/cm)	176.56 ± 2.74 b	173.61 ± 3.16 b	198.86 ± 3.35 a
Ca exch. (mg/kg)	3131.97 ± 92.51 b	3488.06 ± 64.90 b	3884.48 ± 85.60 a
K exch. (mg/kg)	102.85 ± 10.52 c	119.96 ± 11.07 b	182.60 ± 11.27 a
Mg exch. (mg/kg)	178.87 ± 13.15 c	185.36 ± 11.32 b	235.35 ± 6.14 a
Na exch. (mg/kg)	16.43 ± 0.53 a	12.03 ± 0.85 b	17.69 ± 0.53 a

N_tot_ = total nitrogen; C_tot_ = total carbon; C_org_ = organic carbon; DM = dry matter; Olsen P = Olsen phosphorus; EC = electrical conductivity; Ca exch. = exchangeable calcium; K exch. = exchangeable potassium; Mg exch. = exchangeable magnesium; Na exch. = exchangeable sodium.

Soil total nitrogen (N_tot_) and soil organic carbon (C_org_) differed significantly among treatments (p < 0.01): N_tot_ being 26% higher in STV2 than in STV1 and 34% higher than in CI; and C_org_ being 21% higher, on average, in STV2 than in STV1 and CI. Conversely, total carbon (C_tot_) content in STV2 was markedly lower, with reductions of 26% and 30% compared to STV1 and CI, respectively. DM did not show a tendency; however, it was 1% lower in STV1 than CI, and this difference was significant. Olsen phosphorus (Olsen P) was more than double in STV2 compared to STV1 (+102%) and 60% higher relative to CI. Electrical conductivity (EC) increased under STV2 compared to both STV1 and CI (+15% and +24%, respectively) (Table 1).

Regarding exchangeable bases, STV2 consistently displayed elevated levels of key cations: exchangeable calcium (Ca²⁺) was 11% and 24% higher than in STV1 and CI, respectively; potassium (K⁺) increased by 52% compared to STV1 and by 78% relative to CI; and magnesium (Mg²⁺) appears higher by 27% and 32% in comparison to STV1 and CI, respectively (Table 1).

Elemental composition showed significant changes in elemental concentrations across CI, STV1, and STV2, with STV2 exhibiting the highest enrichment for most elements. One-way ANOVA followed by Tukey’s HSD test identified statistically significant increases in 23 out of the 26 measured elements in STV2 compared to both CI and STV1 (Table 2). On average, STV2 showed a 24% increase in elemental concentrations relative to both CI and STV1. The most notable increases were observed for copper (+46% vs CI; +39% vs STV1), potassium (+30%; +29%), molybdenum (+33%; +36%), zinc (+32%; +34%), and lead (+34%; +32%). In contrast, calcium, magnesium, and strontium concentrations were significantly reduced in STV2 (Ca: −43% vs CI; Mg: −18%; Sr: −30%).

**Table 2:** Elemental composition of soils across different conditions. Values are expressed as mean ± standard error (n = 9). CI: control; STV1: standard trackers; STV2: extended trackers. Different letters indicate a statistically significant difference according to the post-hoc test (p < 0.01) (S2 Table in S1 File).

Parameter (mg/kg)	CI	STV1	STV2
Al	28824.97 ± 1779.44 b	30366.15 ± 1392.92 b	38857.50 ± 396.43 a
As	8.97 ± 0.46 b	9.39 ± 0.37 b	11.27 ± 0.1 a
B	17.90 ± 1.08 b	18.60 ± 0.91 b	23.25 ± 0.12 a
Ba	131.56 ± 7.07 b	137.41 ± 5.43 b	173.59 ± 1.71 a
Be	0.96 ± 0.05 b	1.01 ± 0.04 b	1.27 ± 0.01 a
Ca	96353.09 ± 7473.99 a	89179.39 ± 6942.84 a	53456.21 ± 3086.78 b
Cd	0.19 ± 0.01 b	0.19 ± 0.01 b	0.23 ± 0.00 a
Co	8.65 ± 0.45 b	9.11 ± 0.36 b	11.00 ± 0.12 a
Cr	40.48 ± 2.17 b	42.66 ± 1.75 b	53.23 ± 0.51 a
Cu	23.79 ± 2.54 b	26.85 ± 3.08 b	37.27 ± 0.40 a
Fe	19014.43 ± 1133.90 b	19938.86 ± 848.69 b	25348.42 ± 272.66 a
K	6029.93 ± 356.46 b	6354.32 ± 288.67 b	8195.15 ± 92.76 a
Li	26.21 ± 1.35 b	27.76 ± 1.07 b	33.69 ± 0.21 a
Mg	15277.28 ± 442.19 a	14648.3 ± 505.32 a	12521.72 ± 72.45 b
Mn	467.47 ± 22.18 b	496.56 ± 18.89 b	599.46 ± 9.25 a
Mo	0.79 ± 0.04 b	0.80 ± 0.03 b	1.09 ± 0.05 a
Na	473.72 ± 8.44 b	475.67 ± 5.89 b	529.72 ± 7.26 a
Ni	25.46 ± 1.52 b	26.63 ± 1.10 b	33.13 ± 0.31 a
P	634.32 ± 41.05 b	652.07 ± 29.31 b	837.18 ± 10.11 a
Pb	13.49 ± 1.207 b	14.66 ± 1.02 b	19.35 ± 0.11 a
S	238.89 ± 8.27 b	246.04 ± 6.69 b	280.53 ± 2.81 a
Sn	4.19 ± 0.16 b	4.40 ± 0.09 b	4.97 ± 0.05 a
Sr	150.45 ± 7.68 a	142.83 ± 7.18 a	103.40 ± 3.06 b
Ti	1022.48 ± 38.46 b	1051.16 ± 22.56 b	1201.06 ± 27.03 a
V	50.30 ± 2.37 b	52.38 ± 1.77 b	63.80 ± 0.47 a
Zn	51.55 ± 3.87 b	50.97 ± 2.71 b	68.15 ± 0.30 a

**Fig 1:**
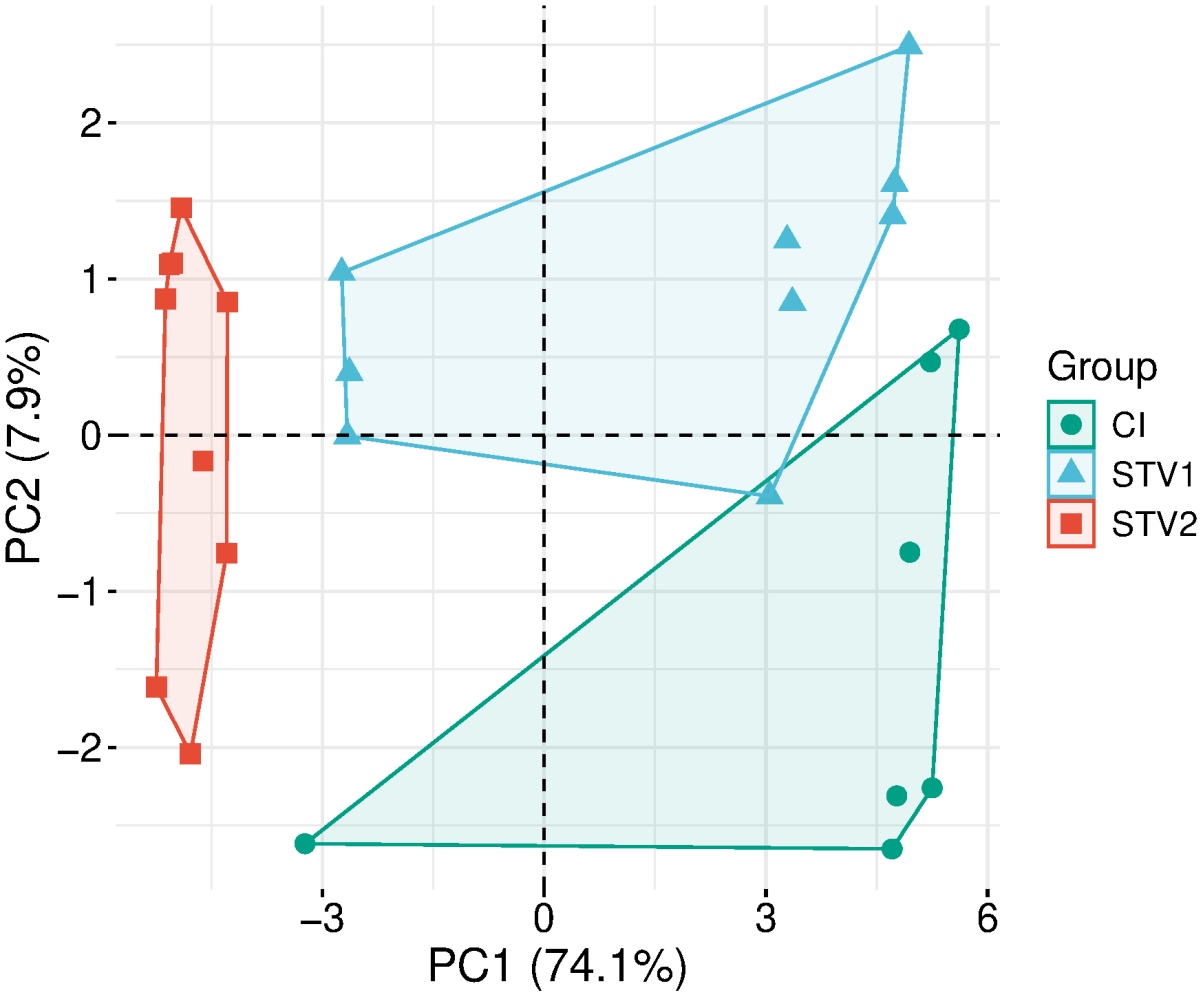
Principal coordinate analysis (PCoA) of soil characteristics based on physicochemical and elemental composition. Shapes represent samples, colored by experimental conditions (STV2, STV1, CI). Axes show the percentage of variance explained by each principal coordinate.

Principal Coordinate Analysis (PCoA; Fig 1) showed that STV2 samples clustered separately along PC1, which explained 74% of the variance. STV1 and CI samples displayed only limited overlap and remained relatively close but distinguishable, with PC2 explaining a substantially smaller proportion of the variance (7.9%). Overall, the ordination indicates a clear separation of STV2, whereas differentiation between CI and STV1 is more subtle (Fig 1).

### 3.2. Microbial community structure

#### 3.2.1. Bacterial and fungal alpha diversity.

To assess the independent and interactive effects of condition and sampling time on microbial composition, the two-way PERMANOVA was performed on the 30 most abundant Taxa using the Bray-Curtis Method with 9999 permutations. For the bacterial community, sampling time had a significant influence (p = 0.0006), but neither the condition nor the interaction between factors were significant (S3 Table in S1 File). In contrast, for the fungal community, although both ampling time and condition were statistically significant (p = 0.0001), the interaction between factors was not significant (S4 Table in S1 File).

Microbial alpha diversity was influenced by both experimental condition and sampling time, with distinct patterns observed for bacterial and fungal communities.

**Fig 2:**
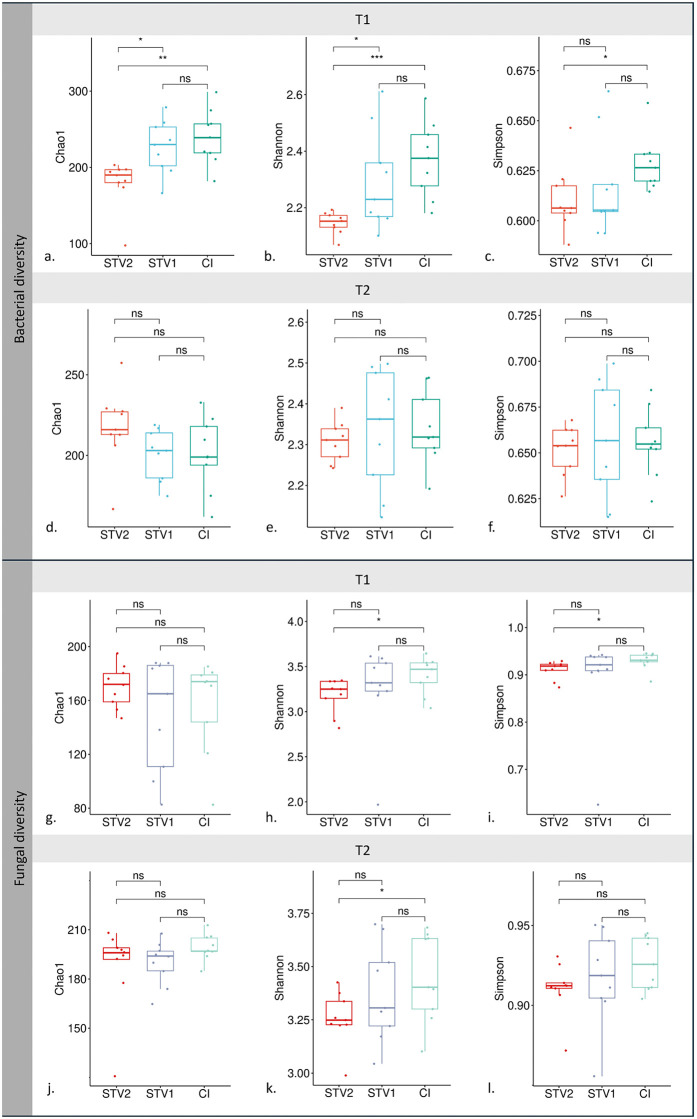
Alpha diversity of microbial communities measured using Chao1, Shannon, and Simpson indices. Panels show bacterial diversity at the genus level under different agrivoltaic (AV) configurations (STV2, STV1) and control (CI), at T1 (**A–C**) and T2 (**D–F**), and fungal diversity at T1 (**G-I**) and T2 (**J-L**). Significance levels: ns (not significant), *p < 0.1, **p < 0.05, ***p < 0.01 (Kruskal-Wallis test). Boxplots represent median and interquartile ranges.

At T1, bacterial alpha diversity was significantly reduced in STV2. Specifically, Chao1 and Shannon indices were significantly lower in STV2 compared to the control (CI) (p < 0.01 and p < 0.05, respectively), and the Simpson index was significantly reduced relative to both CI (p < 0.01) and STV1 (p < 0.05) (Fig 2A–2C). In contrast, no significant differences in bacterial alpha diversity were observed among conditions at T2 for any of the diversity indices considered (Fig 2D–2F).

Fungal alpha diversity showed a different pattern. The Chao1 index did not differ significantly among conditions at either sampling time (Fig 2G, 2J). However, the Shannon index was significantly lower in STV2 compared to CI, at both T1 and T2 (p < 0.05) (Fig 2H, 2K). Similarly, the Simpson index was significantly lower in STV2 than in CI at T1 (p < 0.05), whereas no significant differences were observed at T2 (Fig 2I, 2L).

#### 3.2.2. Bacterial and fungal beta diversity.

**Fig 3:**
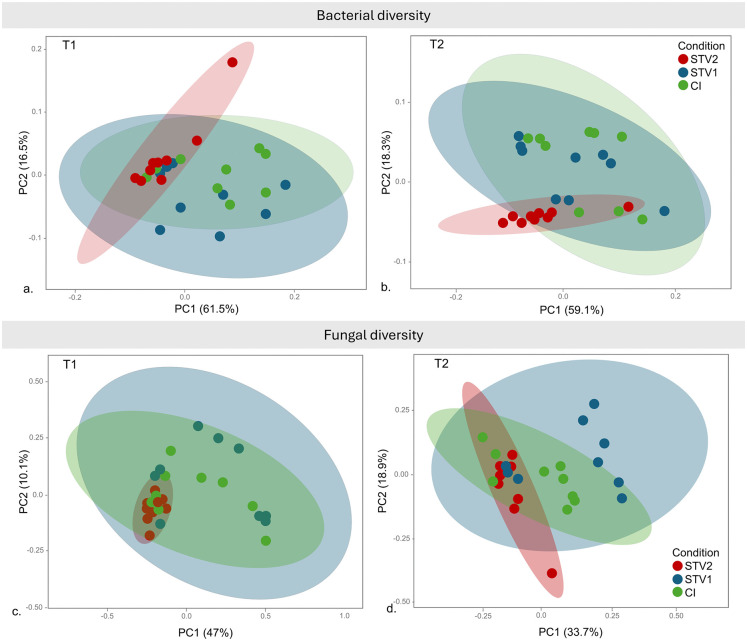
Principal Coordinates Analysis (PCoA) of community composition under AV configurations (STV2, STV1) and control (CI). Panels show bacterial (A, B) and fungal (C, D) community composition at the genus level, at T1 (A, C) and T2 (B, D) sampling time points. Points represent samples; ellipses (if present) indicate 95% confidence intervals per condition.

PCoA indicated condition-related differences in both bacterial and fungal community structures at T1 and T2 (Fig 3). For the bacterial community, the condition explained a significant portion of the observed variation in community composition at T1 (p = 0.0146, R² = 0.23) and T2 (p = 0.0009, R² = 0.33), with STV2 consistently differing from both STV1 and CI (p < 0.01). No significant differences were detected between STV1 and CI (Fig 3A, 3B). Similarly, the fungal community composition was significantly influenced by the condition, which explained a substantial proportion of the variation at both time points (p = 0.0006; R² = 0.39 at T1 and R² = 0.35 at T2) (Fig 3C, 3D). All pairwise comparisons are statistically significant: STV2 vs STV1, STV2 vs CI (p < 0.01), and STV1 vs CI (p < 0.05).

#### 3.2.3. Bacterial and fungal taxonomic composition.

Amplicon sequencing of 16S rRNA and ITS1 regions revealed taxonomically distinct bacterial and fungal communities, whose composition was significantly influenced by condition and sampling time.

**Fig 4:**
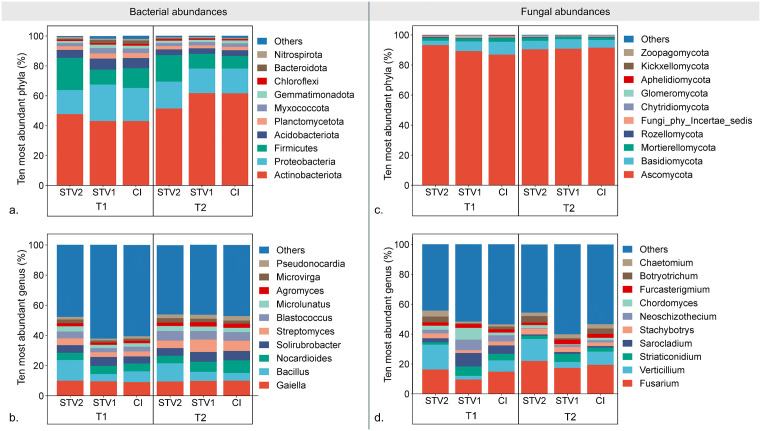
Relative abundance of the 10 most dominant bacterial (A, B) and fungal (C, D) phyla (A, C) and genera (B, D). The figure shows microbial communities in soils under AV configurations (STV2, STV1) and control (CI), at T1 and T2.

Bacterial community composition at the phylum level was dominated by Actinobacteriota (51%), Proteobacteria (19%), Firmicutes (13%), and Acidobacteriota (5%) (Fig 4A). While the relative abundance of several dominant phyla, including Proteobacteria, Acidobacteriota, Planctomycota, Myxococcota, Chloroflexi, and Bacteroidota, remained relatively stable across conditions and sampling times (S5 Table in S1 File), other groups exhibited clear condition- and time-point-specific dynamics. Actinobacteriota increased significantly at T2 compared to T1 (+65%), whereas Firmicutes were consistently more abundant under the extended trackers (STV2), with this difference reaching statistical significance at T2 (+130%). In contrast, Nitrospirota showed higher relative abundance in the control (CI) at T1 but declined significantly at T2 (−50%).

At the genus level, bacterial communities were dominated by *Gaiella*, *Bacillus*, *Nocardioides*, and *Solirubrobacter*, collectively accounting for approximately 10%, 8%, and 6% of the total community, respectively (Fig 4B). All ten most abundant genera showed a significant increase from T1 to T2 time points, with an average relative abundance rise of 77%. The most pronounced increases were observed for *Blastococcus* (+122%), *Streptomyces* (+132%), and *Pseudonocardia* (+133%). *Bacillus* was significantly more abundant in STV2 soils at both time points, with increases of +84% at T1 and +154% at T2, compared to the average of the other two conditions. *Blastococcus*, *Microlunatus*, and *Microvirga* showed significant enrichment in STV2 soil at T2 only (+34%, +39%, and +58%, respectively; S6 Table in S1 File).

At the phylum level, fungal community composition was overwhelmingly dominated by Ascomycota, which accounted for approximately 90% of total sequences among all samples (Fig 4C). No statistically significant differences were detected in phylum-level fungal composition across conditions or sampling times (S7 Table in S1 File).

At a finer taxonomic resolution, the dominant fungal genera included *Fusarium* (17%), *Verticillium* (9%), *Striaticonidium* (4%), and *Sarocladium* (3%) (Fig 4D). Seasonal trends were evident, with genera such as *Fusarium*, *Verticillium*, *Stachybotrys*, *Furcasterigmium*, *Botryotrichum*, and *Chaetomium* increasing in abundance from T1 to T2 (+32% on average). In contrast, *Striaticonidium*, *Sarocladium*, *Neoschizothecium*, and *Chordomyces* decreased by an average of 59%. Although significant differences were detected among conditions for some genera, no consistent trend was observed. Notably, the intermediate configuration (STV1) did not consistently fall between the control (CI) and the extended trackers (STV2) in terms of genus abundance. Instead, the intermediate configuration (STV1) represented the condition under which peak values, either highest or lowest, were recorded for specific genera: *Striaticonidium* reached its statistically highest abundance, whereas *Botryotrichum* exhibited its lowest abundance under STV1, suggesting non-linear, condition-specific responses. Among the ten most abundant genera, *Stachybotrys*, *Chordomyces*, *Furcasterigmium*, and *Chaetomium* did not exhibit significant variation among either condition or sampling time (S8 Table in S1 File).

**Fig 5:**
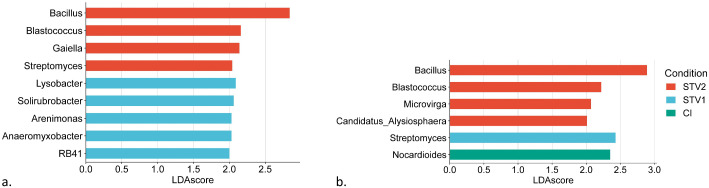
Linear discriminant analysis effect size plots on Bacteria. The analysis shows bacterial genera significantly enriched in STV2 at T2 (LDA > 2; FDR ≤ 0.05).

LEfSe analyses identified *Bacillus* and *Blastococcus* as bacterial genera significantly associated with the STV2 condition, only at T1 (LDA > 2, FDR ≤ 0.05) (Fig 5).

**Fig 6:**
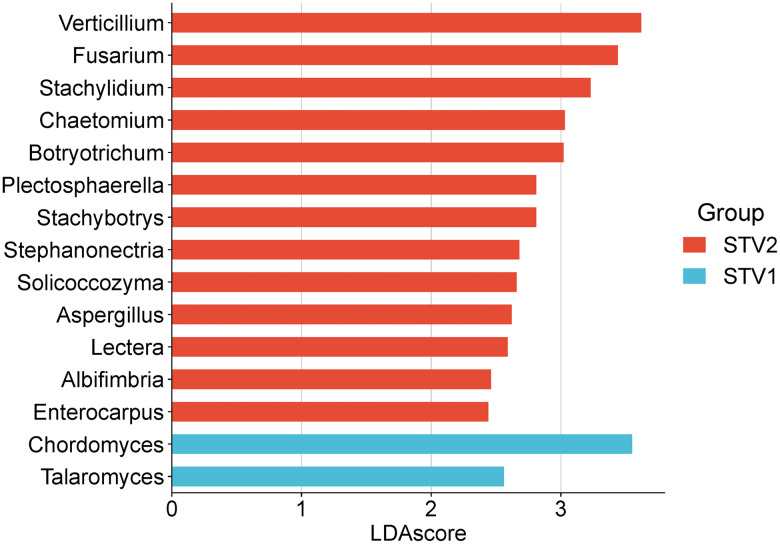
Linear discriminant analysis effect size plots on Fungi. The analysis shows fungal genera significantly enriched in STV2 (red), STV1 (blue), and CI (green) conditions, at T1 (a), and T2 (b) (LDA > 2; FDR ≤ 0.05).

For Fungi, LEfSe analyses identified genera significantly associated with the different conditions (LDA > 2, FDR ≤ 0.05). At T1, *Verticillium* and *Fusarium* emerged as associated with the STV2 AV configuration, *Phaeosphaeriaceae* (*Inc. sedis*) was associated with STV1, while no fungal genera were associated with the control (Fig 6a). At T2, *Botryotrichum* and *Cladorrhinum* were associated with STV2, *Talaromyces* and Rhizoctonia with STV1, and *Lectera* with the control (Fig 6b)

### 3.3. Environmental structuring of microbial communities

To investigate the relationship between soil abiotic factors and microbial community composition, Canonical Correspondence Analysis (CCA) was performed separately for bacterial and fungal communities.

**Fig 7:**
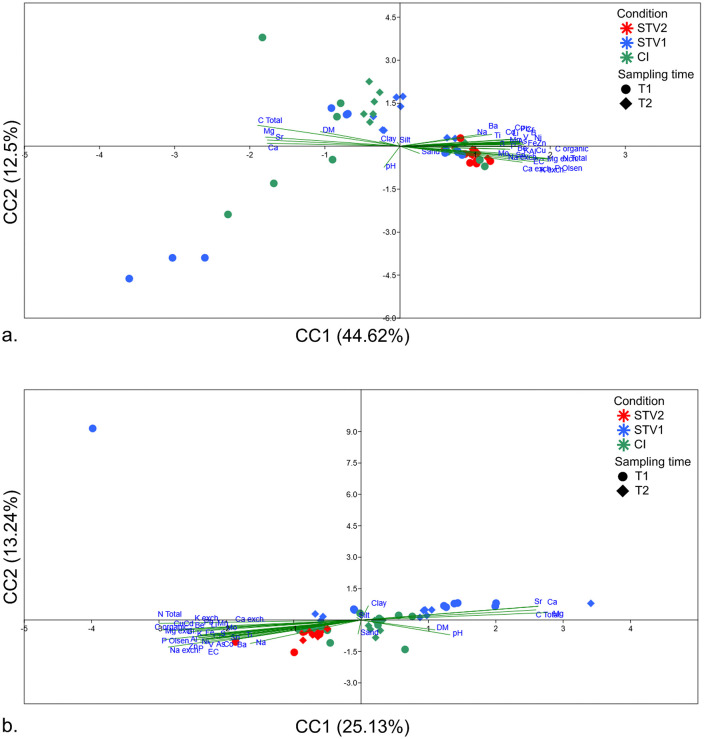
Canonical Correspondence Analysis (CCA) plots showing the relationships between microbial communities and soil chemical-physical and elemental composition. Soil bacterial (A) and fungal (B) communities constrained by soil properties at two time-points: T1 (●), and T2 (♦), in the different conditions: STV2, STV1, CI.

Bacterial communities exhibited moderate environmental structuring when constrained by physicochemical soil properties and elemental composition (Fig 7A). The first two CCA axes explained 57% of the constrained variation (CCA1: 45%, CCA2: 13%), corresponding to 22% of the constrained inertia (S9 Table in S1 File), indicating moderate model fit and limited environmental explanation of bacterial community variation. The ordination showed a tendency for CI and shaded soils (STV1, STV2) to cluster along CCA1, although separation among groups remained partial and not defined. Among the measured variables, C_org_, N_tot_, P Olsen, EC, and exchangeable cations were positively correlated with the shaded plots, STV2 in particular; whereas C_tot_, Mg, Sr, and Ca were more closely associated with the control. Soil texture parameters and pH showed no clear association with the ordination gradients. No clustering related to sampling time was observed.

Fungal communities exhibited weak environmental structuring when constrained by physicochemical soil properties and elemental composition (Fig 7B). The first two CCA axes accounted for 38% of the constrained variance (CCA1: 25%, CCA2: 13%), corresponding to 14% of the total inertia (S10 Table in S1 File), indicating a limited environmental explanation of fungal community variability. Overall, the ordination did not show distinct grouping among conditions: STV2 samples tended to cluster, which was mostly driven by C_org_, N_tot_, P Olsen, EC, and exchangeable cations. Again, no clustering related to the sampling time was observed.

## 4. Discussion

Integrating photovoltaic systems into agricultural fields was associated with changes in soil conditions and with shifts in microbial community composition.

### 4.1. Effects of AV systems on soil physicochemical properties

Differences in nitrogen and carbon dynamics across treatments suggest that AV shading may influence the balance between mineralization and immobilization processes. The lower C/N ratio in STV2 soils suggests greater nitrogen retention than in CI and STV1 soil conditions. These trends may reflect shifts in organic matter decomposition and microbial turnover rates potentially linked to altered vegetation and soil–water interactions beneath the panels. Although microclimatic parameters were not measured, the lower dry matter content under panels is indicative of greater soil moisture retention (Table 1). These observations align with Luo et al. [41], who reported minimal effects on soil pH, slight increases in N_tot_, and significant enhancements in Olsen P and EC under AV systems. While Luo et al. [41] focused on legume-based systems, we observed a comparable increase in N_tot_ under wheat cultivation, suggesting that N accumulation under AV systems may not be solely dependent on biological fixation but also on changes in soil processes. The increases in available P, EC, and exchangeable base cations (Ca^2+^, K^+^, Mg^2+^) under STV2 indicate a tendency toward increased nutrient retention in shaded soils (Table 1). Because all plots were uniformly fertilized, reduced exposure to direct rainfall beneath the panels may have limited nutrient leaching, allowing fertilizers and dissolved ions to accumulate in the upper soil layers. This accumulation could increase nutrient availability and modify the balance of exchangeable cations. The slight shift from a Ca-dominant profile in CI toward a higher K saturation in STV2 is consistent with the concept that reduced Ca dominance can favour nutrient uptake and early plant growth [42], although this relationship should be interpreted cautiously. Elemental profiling further supported this pattern: STV2 soils were enriched in 23 out of 26 measured elements (Table 2), suggesting increased accumulation of micronutrients and trace elements under shaded conditions. This enrichment may reflect altered water-solute dynamics caused by panel shading [43]. In the absence of direct precipitation, but under continued plant transpiration, water can be drawn upward [44], a process known to promote ion accumulation in surface horizons [45]. The observed increase in exchangeable Ca²⁺ and Mg²⁺ , without a corresponding rise in total Ca and Mg, is consistent with this interpretation, a redistribution of nutrients toward the biologically active soil layer. Interestingly, total Ca in STV2 soil was markedly lower than in the control (Table 2). This suggests that capillary transport may have promoted carbonate solubilization and CO₂ release. Changes in exchangeable and total calcium may be linked to carbonate dissolution processes, but this mechanism requires further verification [46]. In the same context, the increase in C_org_ together with the decrease in C_tot_ suggests a decoupling between organic and inorganic carbon pools. While shading may have promoted organic matter accumulation, the reduction in total carbon may reflect a decrease in the inorganic (carbonate) fraction, possibly linked to altered soil moisture and microclimatic conditions under the panels. This interpretation offers a plausible explanation for the chemical data, though alternative hypotheses remain possible. Previous studies similarly reported that shading and reduced wind speed under PV systems decrease soil evaporation [13], and that vegetation under the panels can lower soil surface temperatures [47,48]. Although we did not directly measure moisture or temperature, the observed accumulation of C_org_ and nutrients in STV2 is consistent with the hypothesis that moderated microclimatic conditions may contribute to improved soil functioning in AV systems [49].

Taken together, these observations suggest that the physicochemical changes observed in STV2 soils likely arise from multiple interacting processes, including shading, modified water–solute interactions, and vegetation cover. Rather than representing a direct effect of shading alone, the results point to the AV system as a whole as a potential driver of these soil changes. The higher ground coverage ratio (STV2) was associated with enhanced nutrient retention and organic matter accumulation although the relative contribution of individual mechanisms remains to be determined.

### 4.2. Effects of AV systems on microbial diversity and community

Bacterial diversity was lower in STV2, and this pattern coincided with a higher abundance of copiotrophic genera such as *Bacillus* (Fig 1A–1C). The reduction in alpha diversity was primarily driven by the increased abundance of this genus, which reduced community evenness. Such nutrient-driven dominance patterns have often been documented in soil microbiomes, where shifts in resource availability can alter both taxonomic composition and functional potential [50]. The effect was more evident in Shannon and Simpson indices (dominance), than in Chao1 (richness). The relative abundances of the ten most common genera (excluding *Bacillus*) remained broadly comparable across treatments, suggesting that the decrease in diversity was linked more to shifts in relative dominance than to the loss of rare taxa. *Bacillus* is a copiotrophic and fast-growing genus that thrives in nutrient-rich environments [51]. Its proliferation in STV2 soils is consistent with the elevated levels of C_org_, Olsen P, EC, and N_tot_ observed in this condition. The combination of enhanced nutrient availability and dense vegetation may have favoured opportunistic taxa capable of rapid resource exploitation, reducing overall evenness and thereby lowering Shannon and Simpson diversity. At T2, although *Bacillus* remained more abundant in STV2 compared to STV1 and CI, no significant decrease in alpha diversity was detected according to any of the indices considered (Fig 2D–2F). This may reflect the concurrent increase in *Blastococcus*, *Microlunatus*, and *Microvirga*, which mitigated the dominance effect by enhancing the community’s evenness.

Fungal communities showed smaller changes in diversity, remaining relatively stable across treatments, consistent with their slower turnover and substrate dependence. This reduction in evenness corresponded to an increased abundance of *Verticillium* in STV2, approximately twice that observed in CI and about seven times higher than in STV1. While not all comparisons were statistically significant, this pattern suggests a possible response of communities to nutrient enrichment through selective stimulation of a few opportunistic taxa. At T2, only the Shannon index retained a similar trend (Fig 2L–2N), possibly reflecting seasonal changes or shifts in substrate availability.

Overall, the panel appeared to have a stronger association with ecological indices at T1 than at T2, which may be explained by the shorter shadows cast at T2 (summer), resulting in less pronounced microclimatic differences. This temporal variability may reflect a stronger influence of stochastic processes and dispersal dynamics on bacterial than on fungal assemblages [52]. Bacterial communities showed clearer seasonal and treatment-related shifts (Fig 3A, 3B), reflecting rapid responses to environmental fluctuations and nutrient dynamics, whereas fungal communities (Fig 3C, 3D) remained relatively stable, supporting the view that fungi are more closely linked to persistent habitat features such as soil structure and organic matter pools [53–56]. This stability is consistent with the perspective of fungi as mediators of long-term soil process [57]. The reduction in microbial diversity under STV2 is consistent with the ecological principle that high resource availability can favour competitive dominance of fast-growing taxa, potentially reducing evenness [58,59].

LEfSe analysis revealed distinct microbial taxa significantly enriched in each condition, indicating that AV configurations exert consistent selective pressures on soil microbial communities. Across both bacterial and fungal datasets, STV2 consistently exhibited the highest number of enriched taxa, suggesting a community shaped by strong deterministic selection linked to nutrient redistribution beneath the panels.

In STV2 soils, *Bacillus* and *Blastococcus* emerged as associated with the condition at T2 (Fig 5A, 5B). Their prevalence reflects nutrient enrichment and microclimatic buffering under high shade: *Bacillus*, a copiotrophic genus with plant growth-promoting traits [51,60], thrives in resource-rich soils, while *Blastococcus*, typically stress-tolerant, likely benefits from metabolic versatility and possible root-associated interactions [61]. STV1 and CI did not show any associated genera, at either T1 or T2.

For fungi, differential taxa were mainly detected at T1 (Fig 6a) and were mostly associated with STV2. These included *Verticillium*, and *Fusarium*, consistent with their preference for moist, nutrient-enriched habitats [62–64]. These genera are common soilborne taxa that can exploit shaded microsites with episodic increases in resource availability, especially nitrogen and labile carbon [65–67]. Their enrichment under STV2 suggests that this configuration fosters ecological conditions favoring the persistence of opportunistic taxa. Under moderate shading (STV1), *Phaeosphaeriaceae* prevailed, as it is generally classified as saprobic and commonly isolated from soils [68]. At T2, specific fungal indicator taxa were identified across the different conditions (Fig 6b). STV2 was mainly associated with *Botryotrichum* and *Cladorrhinum*, two soil-associated genera commonly reported in organic-matter–rich environments and frequently linked to saprotrophic activity [69–71]. Several taxa were instead linked to STV1, including *Talaromyces* and *Rhizoctonia*. *Talaromyces* is a ubiquitous genus known for producing a wide range of secondary metabolites and for its potential role in biocontrol [72], whereas *Rhizoctonia* includes often pathogenic species widely distributed in agricultural soils and involved in root–soil interactions [73]. Their presence under STV1 may reflect conditions of moderate shading and nutrient availability that favour fungi capable of exploiting diverse organic substrates. *Lectera* was associated with the control soils and has been reported as a patotroph, but its specific relationship with soil fertilizers remains poorly understood [74]. Overall, this shift suggests a reduced differentiation among treatments over time and a more heterogeneous distribution of fungal indicator taxa*.*

### 4.3. Environmental drivers of community assembly

The Canonical Correspondence Analyses (CCA) revealed partially differentiated patterns in the environmental structuring of bacterial and fungal communities in response to soil physicochemical and elemental composition. The first canonical axis (CCA1) accounted for the majority of the constrained variance. Accordingly, the interpretation of community-environment relationships was primarily guided by the distribution of samples and environmental vectors along CCA1. Sample dispersion patterns varied, suggesting that bacterial and fungal communities may respond differently to environmental gradients.

For bacteria, the results suggest that a limited but non-negligible proportion of community variation was associated with the measured soil variables. This suggests a partial environmental influence rather than strong deterministic control, whereby certain physico-chemical gradients may contribute to shaping bacterial community patterns. This pattern may reflect the ability of specific bacterial taxa to respond to key physico-chemical drivers such as pH, nutrient availability, and element concentrations, despite the overall compositional flexibility and rapid turnover of bacterial communities. Unmeasured factors, such as micro-scale heterogeneity, root exudates, redox conditions, and microbial interactions, may explain the remaining variation [75–79].

In contrast, fungal communities were only weakly structured by the same environmental variables, with just 14% of the total inertia constrained by the CCA model. This suggests that unmeasured or more stochastic factors, such as plant host identity, substrate availability, spatial dispersal limitations, or legacy effects, may influence the fungal assemblages in this system. Fungi, particularly mycorrhizal and saprotrophic taxa, are often expected to respond to edaphic gradients [80]; however, in our study, the low explanatory power suggests that such relationships were not captured, potentially due to landscape-level heterogeneity, functional redundancy, or the buffering effect of filamentous growth habits.

### 4.4. Functional microbial ecology considerations

An increase in the relative abundance of several fungal genera that include known plant pathogens (e.g., *Verticillium* and *Fusarium*) was observed under the STV2 treatment. This pattern may be consistent with the higher soil moisture conditions likely created by panel shading and reduced evaporative demand. However, these genera include diverse ecological groups, and metabarcoding approaches based on ITS markers do not allow discrimination between pathogenic, endophytic, and saprotrophic species within these taxa. Consequently, the presence or relative enrichment of these genera should be interpreted cautiously and does not provide direct evidence of increased disease risk. In addition, several of the taxa detected (e.g., *Chaetomium, Botryotrichum*) are widely recognized as saprotrophic fungi involved in organic matter decomposition and nutrient cycling. Possible shifts in the balance between saprotrophic and potentially pathogenic fungal guilds under altered microclimatic conditions therefore remain speculative and warrant targeted investigation, particularly in relation to plant health and crop productivity.

These results indicate that AV management was associated with changes in soil nutrient profiles and microbial community structure, even under uniform fertilization and crop management practices. The STV2 configuration showed higher concentrations of C_org_, N_tot_, available P, and exchangeable cations, together with the enrichment of numerous trace elements. These changes were accompanied by measurable shifts in microbial diversity and composition, especially in the bacterial assemblages. Bacterial communities exhibited stronger seasonal and treatment-related variability, whereas fungal assemblages remained comparatively stable across conditions and sampling times. This contrast is consistent with the idea that bacteria may respond more rapidly to short-term changes in soil properties, while fungi may be more strongly associated with persistent edaphic factors such as substrate quality and nutrient pools. However, CCA indicated that measured soil parameters explained only part of the observed variation, suggesting that additional unmeasured factors, potentially biological or spatial, contribute to shaping microbial assemblages. Collectively, these findings provide an initial framework for interpreting how AV systems may influence soil–microbe interaction, highlighting the need for broader evaluation across different designs and environments.

## 5. Conclusions

Our findings indicate that agrivoltaic system design, particularly ground coverage ratio (GCR), is associated with changes in soil biogeochemical properties and microbial community structure. The high-shade configuration (STV2, 41% GCR) was characterized by higer levels of organic carbon, nutrient retention, and exchangeable cations. It was also associated with reduced microbial diversity, favoring copiotrophic taxa such as *Bacillus* and hemibiotrophic plant pathogens, such as *Verticillium*. This may reflect a more selective and stable microenvironment that favours specialized microorganisms, although causal mechanisms remain to be clarified. While shifts in soil chemistry and microbial composition showed general correspondence, their relationship appeared less pronounced at T2, possibly reflecting reduced shading influence. Overall, our results suggest that AV system design may influence belowground processes alongside aboveground productivity and energy efficiency. Future research should further investigate whether AV-associated microbial shifts affect ecosystem services such as carbon sequestration and pathogen suppression, for example through functional metagenomics and trait-based approaches. Although these findings are specific to wheat-based systems in temperate climates, they underscore the importance of evaluating belowground impacts across diverse crop types and environmental conditions when designing agrivoltaic systems, to ensure both agronomic and ecological sustainability.

## Supporting information

S1 File Contains S1–S10 Tables. One table for each page of the file.
(XLSX)
